# Oviduct Fluid Extracellular Vesicles Change the Phospholipid Composition of Bovine Embryos Developed In Vitro

**DOI:** 10.3390/ijms21155326

**Published:** 2020-07-27

**Authors:** Charles Banliat, Daniel Le Bourhis, Ophélie Bernardi, Daniel Tomas, Valérie Labas, Pascal Salvetti, Benoît Guyonnet, Pascal Mermillod, Marie Saint-Dizier

**Affiliations:** 1 INRAE, CNRS, University of Tours, IFCE, UMR 85 PRC, F-37380 Nouzilly, France; charles.banliat@inrae.fr (C.B.); ophelie.bernardi@inrae.fr (O.B.); daniel.tomas@inrae.fr (D.T.); valerie.labas@inrae.fr (V.L.); pascal.mermillod@inrae.fr (P.M.); 2 Union Evolution, F-35530 Noyal-Sur-Vilaine, France; benoit.guyonnet@evolution-xy.fr; 3 Allice, F-37380 Nouzilly, France; daniel.lebourhis@allice.fr (D.L.B.); pascal.salvetti@allice.fr (P.S.); 4 INRAE, Université de Tours, CHU de Tours, Plate-forme CIRE, F-37380 Nouzilly, France; 5 Department Agrosciences, Faculty of Sciences and Techniques, University of Tours, F-37200 Tours, France

**Keywords:** extracellular vesicles, exosomes, microvesicles, tubal fluid, fallopian tube, oviduct, embryo, bovine, cattle, lipidomics

## Abstract

Oviduct fluid extracellular vesicles (oEVs) have been proposed as bringing key molecules to the early developing embryo. In order to evaluate the changes induced by oEVs on embryo phospholipids, fresh bovine blastocysts developed in vitro in the presence or absence of oEVs were analyzed by intact cell MALDI-TOF (Matrix assisted laser desorption ionization—Time of flight) mass spectrometry (ICM-MS). The development rates, cryotolerance, and total cell number of blastocysts were also evaluated. The exposure to oEVs did not affect blastocyst yield or cryotolerance but modified the phospholipid content of blastocysts with specific changes before and after blastocoel expansion. The annotation of differential peaks due to oEV exposure evidenced a shift of embryo phospholipids toward more abundant phosphatidylcholines (PC), phosphatidylethanolamines (PE), and sphingomyelins (SM) with long-chain fatty acids. The lipidomic profiling of oEVs showed that 100% and 33% of the overabundant masses in blastocysts and expanded blastocysts, respectively, were also present in oEVs. In conclusion, this study provides the first analysis of the embryo lipidome regulated by oEVs. Exposure to oEVs induced significant changes in the phospholipid composition of resulting embryos, probably mediated by the incorporation of oEV-phospholipids into embryo membranes and by the modulation of the embryonic lipid metabolism by oEV molecular cargos.

## 1. Introduction

Understanding how the mother communicates with the early developing embryo is a scientific challenge. In ruminants as in other mammals, the oviduct is the first environment to which the embryo is exposed [1,2]. The oviductal fluid is a complex fluid containing proteins, carbohydrates, lipids, and ions that are regulated in abundance throughout the estrous cycle in domestic mammals [3]. Recently, extracellular vesicles from the oviductal fluid (oEVs), also called oviductosomes, have been proposed as key participants of the early embryo–maternal dialog [4,5]. EVs are lipid bilayer-enclosed nanosized vesicles, including exosomes (40–100 nm) and microvesicles (100–1000 nm) [6]. As EVs in other biological fluids, oEVs contain a range of biomolecules such as proteins, small RNAs, metabolites, and genomic DNA [4,7,8,9]. Using fluorescent-labelled oEVs and confocal microscopy, it was shown that bovine blastocysts were able to take up oEVs added to the culture medium and internalize them inside their blastomeres [10]. Furthermore, the supplementation of culture media with oEVs has been shown to improve the quality of bovine blastocysts in terms of total cell number, cryotolerance, and hatching rates [10,11,12]. Exposure to oEVs was also shown to induce changes in gene expression, miRNA content, and global DNA methylation levels in cattle embryos [9,11,12]. In mice, oEVs from donor oviduct fluid in the transfer medium were shown to improve birth rates after transfer of in-vitro-produced embryos to recipient mothers [13].

Phospholipids, including phosphatidylcholines (PC), phosphatidylethanolamines (PE), and sphingomyelins (SM), represent key structural components of cell membranes and also major components of EVs [14,15]. Glycerophospholipids and sphingolipids are involved in a wide range of cell-signaling pathways and act as precursors to many biomolecules such as lysophosphatidylcholines (LPC), lysoPE (LPE), and eicosanoids [16]. Using intact-cell MALDI-TOF (Matrix assisted laser desorption ionization—Time of flight) mass spectrometry (ICM-MS), we showed earlier that the bovine oviductal fluid contains a mixture of PC, PE, LPC, LPE, and SM varying in abundance throughout the estrous cycle, and it was suggested that oEVs contribute a large part to these phospholipids [17]. Furthermore, significant differences in various PC and SM were reported between in-vitro-produced cattle blastocysts and their in-vivo conceived counterparts [18], indicating that the maternal environment modulates the phospholipid composition of oocytes and developing embryos.

We hypothesized that oEVs added to the culture medium would change the lipid composition of developing embryos in a way that might explain the beneficial effects of oEVs on embryo quality. The objective of this study was thus to examine the effect of oEVs on the phospholipid composition of bovine blastocysts.

## 2. Results

### 2.1. Oviductal Extracellular Vesicles Had No Effect on Embryo Development

The same experiment was performed in two different laboratories (see Materials and Methods Section and Figure 4). Supplementation with postovulatory oEVs during in vitro culture had no effect on embryo cleavage on day 2 or blastocyst rates from cleaved embryos on days 6, 7, and 8 (Table 1).

The rates of blastocyst hatching on day 8 did not differ between groups (control vs. oEVs: 11.3 ± 3.8% vs. 19.3 ± 4.0%; data from Experiment 2). Supplementation with oEVs had no effect on mean cell number per blastocyst on day 8 (85.4 ± 5.3 vs. 97.3 ± 9.8; n = 22 and n = 12, respectively) but tended to increase this number in expanded blastocysts (133.1 ± 5.7 vs. 146.6 ± 5.2; n = 47 and n = 69, respectively; *p* = 0.09; data from Experiment 1).

After slow freezing and thawing, blastocyst re-expanding (control vs. oEVs: 100% vs. 94.7% and 94.7 vs. 84.2% at 24 and 48 h, respectively) and hatching (control vs. oEVs: 31.6% vs. 57.9% and 84.2% vs. 84.2% at 24 and 48 h, respectively) did not differ between groups (n = 19 blastocysts/group; data from Experiment 1).

### 2.2. Oviductal Extracellular Vesicles Changed Embryo Phospholipid Profiles in a Stage-Specific Manner

The lipidomic analysis of fresh individual blastocysts (n = 25) and expanded blastocysts (n = 53) on day 8 detected a total of 259 peaks in the 350–900 m/z mass range, corresponding mostly after annotation to PC, PE, LPC, and SM (see all masses and corresponding annotations in Supplementary data A).

**Figure 1:**
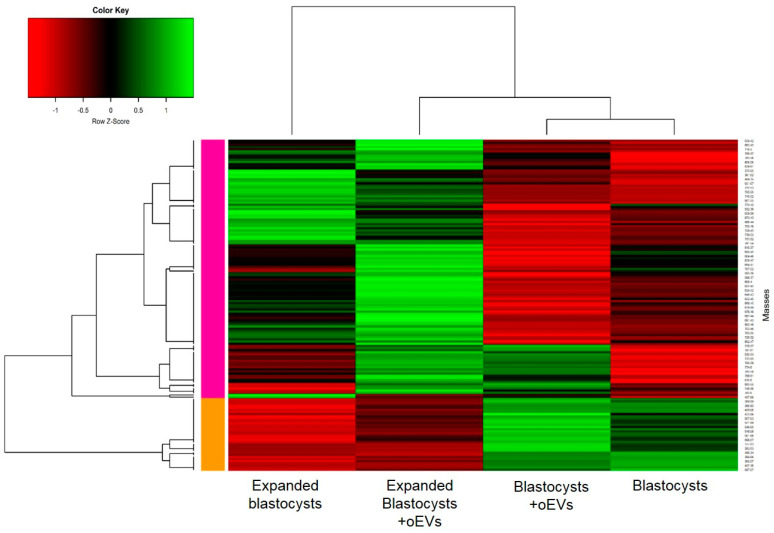
Heatmap representation of hierarchical clustering of the differentially abundant lipid m/z according to the embryonic stage (blastocyst vs. expanded blastocyst) and exposure to oEVs (+oEVs). Each line corresponds to one molecular species. For a given species, green lines represent higher abundance while red lines represent lower abundance compared with other conditions. Black lines represent the median abundance values. The proximity between the conditions and lipid profiles are shown by the hierarchical trees on the top and left of the heatmap, respectively. A total of 25 blastocysts (16 control, 9 oEV-treated) and 53 expanded blastocysts (28 control, 25 oEV-treated) were analyzed.

The hierarchical clustering of differential m/z (*p*-value < 0.05) between oEV-treated and control groups showed a clear separation between blastocysts and expanded blastocysts and evidenced specific lipidomic profiles due to oEV supplementation at each stage (Figure 1; see individual profiles for blastocysts and expanded blastocysts in Supplementary data B).

**Figure 2:**
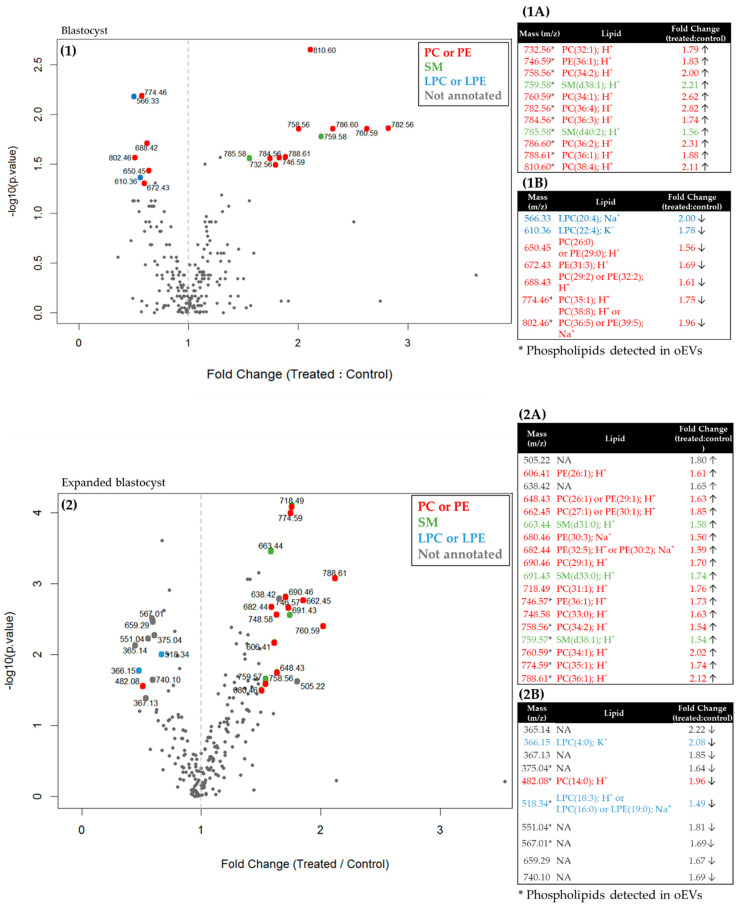
Volcano plots of lipid m/z significantly affected by oEV supplementation in blastocysts (**1**) and expanded blastocysts (**2**) (*p*-value < 0.05; fold-change >1.5 or <0.67). The fold-change of treated vs. control group was plotted against the −log10 *p*-value. Dots in red, orange, and blue indicate significantly altered PC or PE, SM, and LPC or LPE, respectively. Tables on the right indicate the differentially abundant masses that increased (**1A**,**2A**) or decreased (**1B**,**2B**) in oEV-treated embryos compared to controls. PC, phosphatidylcholine; PE, phosphatidylethanolamine; SM, sphingomyelin; LPC, lysophosphatidylcholine; LPE, lysoPE. Note that the first number in parentheses refer to the total number of carbons and the second to the number of double bonds in all chains. * Phospholipids also detected in oEVs (see Section 2.3).

The Volcano plot analysis with a fold-change threshold of 1.5 between oEV-treated and control embryos evidenced 18 and 28 differentially abundant lipid m/z in blastocysts and expanded blastocysts, respectively (Figure 2). In total, 61% (11/18) and 64% (18/28) of differentially abundant m/z were increased in oEV-treated embryos compared with controls in blastocysts and expanded blastocysts, respectively. Among those, six molecular species between 756 and 788 m/z, all overabundant in oEV-treated embryos, were shared between blastocysts and expanded blastocysts and annotated as PE (36:1), PC (34:2), SM (d38:1), PC (34:1), PC (35:1), and PC (36:1). Furthermore, overabundant masses due to oEVs supplementation included a majority of PC, PE, and SM with long-chain fatty acids; whereas LPC/LPE (with one fatty acid group) were identified only among m/z found less abundant following oEV supplementation.

**Figure 3:**
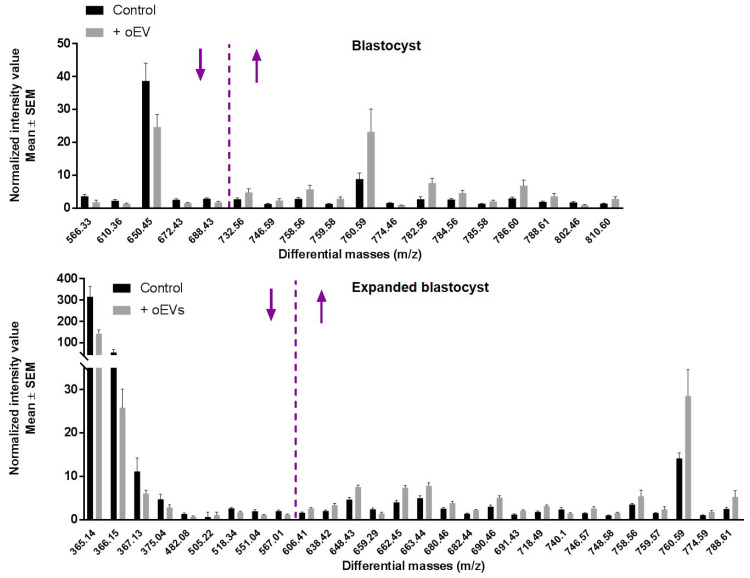
Relative abundance (mean ± SEM) of lipids significantly affected by oEV supplementation in blastocysts (top) and expanded blastocysts (down) and ordered in increasing m/z values (*p*-value < 0.05; fold-change > 1.5 or <0.67). The m/z cut-off at which lower lipid masses were less abundant after oEV supplementation and a majority of higher masses were more abundant is shown by a vertical purple dotted line. See all intensity values and *p*-values in supplementary data A.

Relative abundance of differential masses ordered in increasing *m*/*z* values confirmed that less abundant *m*/*z* in oEV-exposed vs. control embryos included a majority of low lipid *m*/*z*; whereas overabundant masses were almost exclusively phospholipids of high molecular weight, with a cut-off around 700 and 600 *m*/*z* in blastocysts and expanded blastocysts, respectively (Figure 3).

### 2.3. Majority of Overabundant Masses in oEV-Treated Embryos Were Also Detected in oEVs

In order to determine if the m/z found more abundant in oEV-treated compared with control embryos might originate from oEVs, the oEV sample used for in vitro embryo development (postovulatory stage, ipsilateral to ovulation) was analyzed by ICM-MS (6 replicates). A total of 234 lipid peaks in the 350–900 m/z mass range, including mostly LPC, PC, PE, and SM, were detected in oEVs (see details in Supplementary data A). Of the 11 and 18 m/z found increased after oEV exposure in blastocysts and expanded blastocysts, respectively, 11 (100%) and 6 (33%) were also detected in oEVs. The lipid species shared between oEVs and embryos are indicated by an asterisk in Figure 2.

## 3. Discussion

The oviductal fluid is the first interface between the mother and the early developing embryo. Oviduct fluid EVs have been proposed as nanocarriers of molecules for gametes and embryos [4,5,19], but the regulation of the embryonic lipidome by oEVs remains to be explored. Here, for the first time, we show that exposure to oEVs during in vitro culture changed the phospholipid composition of developing embryos in favor of PC, PE, and SM of high molecular masses. Oviductal EVs had specific impact on blastocyst phospholipids before and after blastocoel expansion.

Based on two successive experiments on more than 1500 zygotes, we did not observe any significant improvement in the blastocyst yield after supplementation of the culture medium with oEVs. This is in accord with some previous studies that used oEVs derived from bovine oviduct epithelial cells in vitro [11] and oviductal fluid [12]. In the latter, a detrimental effect of oEVs on blastocyst development was even observed at day 7 that was compensated on days 8 and 9 [12] By contrast, a previous study reported an improvement of blastocyst rates due to oEV supplementation but with a more marked effect on day 9 than on days 7 and 8 [10]. Here, in order to analyze blastocyst quality and phospholipid composition, we stopped embryo development at day 8, preventing us from assessing a potential later effect on embryo development. In the study from Alminana et al. [10] and ours, frozen–thawed oEVs collected from oviductal fluids at the postovulatory stage were used. Furthermore, as previously reported [7], the proportions of exosomes (30–100 nm: 76%) and of microvesicles (100–500 nm: 24%), as measured by transmission electron microscopy in the oEV samples used, were very similar in the two studies. However, the differences between the two studies may be due to differences in oEV content in the culture media: based on preliminary (unpublished) data comparing different doses of oEVs proteins (0.005, 0.05, 0.5 mg/mL) in the culture medium, the 0.05 dose tended to increase the rates of blastocysts at days 7–8 compared to controls without oEVs and was chosen for the present study, whereas Alminana et al. [10] used final concentrations of 0.2–0.4 mg of proteins/mL.

A tendency to increase the total cell numbers in expanded blastocysts after oEV exposure was observed. These results are in line with previously reported positive effects of oEVs on growth and expansion of in-vitro produced cattle embryos [4,10]. Slow freezing in a two-step protocol is currently the method mostly used for cattle embryo cryopreservation in France. We expected a positive effect of oEVs on embryo cryosurvival after slow freezing, as previously reported by others after vitrification [11,12]. However, there was no detectable effect of pre-exposure to oEVs on blastocysts re-expansion and hatching at 24 or 48 h after thawing. It is to note that thawed blastocysts were cultured for 2 days in the presence of 1% of postovulatory cow serum. An uptake of serum EVs added for 24 h in the culture medium was previously shown to occur in bovine blastocysts [20]. It may be that serum EVs masked the possible effects of oEVs during this culture period. It is also worth noting that the survival rates were globally very high (>94% and 84% of re-expansion at 24 and 48 h) in both groups, precluding any evaluation of the effects of oEV in suboptimal conditions. This is probably due to the selection of expanded blastocysts of excellent morphology (grade 1) for cryopreservation. Previous studies examining the effect of liposomes (containing only phospholipids and cholesterol) or of soybean lecithin (mix of phospholipids) on in vitro embryo development have resulted in slight improvements of embryo survival after slow freezing in one [21] but not all [22] studies, and failed to evidence any effect on blastocyst hatching and pregnancy rates after transfer to the uterus of recipient cows [21]. Thus, the effects of oEVs on cattle embryo cryosurvival after slow freezing remains to be evaluated.

The mechanisms involved in cellular communications mediated by EVs include fusion between EV membranes and the plasma membrane of the target cells, as well as EV endocytosis via specific target cell receptors [5]. Previous studies evidenced that oEVs are able to fuse with the membrane of spermatozoa in mice [23] and cats [24] in vitro, leading to the transfer of oEV membrane components to the sperm membrane [23,25]. Previous confocal microcopy observations in our laboratory showed that oEVs added in the culture medium of bovine embryos are able to cross the zona pellucida and localize into the cytoplasm of embryo blastomeres [10]. Similarly, uterine EVs were shown to be internalized by trophectoderm cells of embryos at later stages of development in sheep [26] and humans [27,28]. Based on these observations, we made the hypothesis that the oEVs added to the culture medium are captured by developing embryos, leading to the incorporation of their phospholipids in the plasma membrane and/or cytoplasm of blastomeres. In line with this hypothesis, the majority (>61%) of differentially abundant m/z were overabundant in oEV-exposed embryos compared to controls. In order to evaluate which phospholipids could be brought by oEVs through internalization, we analyzed the lipidomic profile of the pool of oEVs used for the supplementation of the culture medium and detected PC, SM, and PE as major phospholipid species, in accordance with a recent lipidomic analysis of EVs isolated from the uterine fluid of ewes [29]. A perspective of this study is to characterize the phospholipid profiles of oEVs across the cycle with a higher number of replicates. The present analysis enabled us to identify up to 100% (11/11) of overabundant masses in oEV-exposed blastocysts as also present in oEVs, which is consistent with the assumption of an internalization of oEV-derived phospholipids by developing embryos. However, this percentage was much lower (33%, 6/18) after blastocoel expansion. Furthermore, around one third of differentially abundant masses (39% and 36% in blastocysts and expanded blastocysts, respectively) were decreased in embryos after oEV supplementation, indicating that mechanisms other than the simple internalization of oEV-phospholipid cargos inside embryonic cells were involved in these changes. Of note, EVs can carry a large panel of lipid mediators and molecules potentially involved in lipid metabolism including free fatty acids, eicosanoids, proteins, and nucleic acids [15,30]. Previous transcriptomic analyses showed that oEV supplementation during in vitro culture altered the expression of genes involved in lipid metabolism and regulation of lipid metabolic processes in cattle blastocysts [9,12]. Furthermore, various miRNAs involved in fatty acid biosynthesis and metabolism were found highly abundant in bovine oEVs isolated during the periovulatory period compared to the luteal phase of cycle [31]. Therefore, the exposure to oEVs during in vitro culture might have led to both the reorganization of blastomere plasma membrane with oEV-derived phospholipids and to the modulation of embryonic lipid metabolism by small RNAs and proteins brought by oEVs. Further experiments using EV imaging [32] would be necessary to confirm the EV uptake and precisely determine if this uptake varies according to the developmental stage.

The embryos exposed to oEVs displayed a shift toward PC, PE, and SM of higher molecular weight, i.e., possessing fatty acids of longer chains, whereas PC and PE species of lower molecular weight and LPC were detected among less abundant phospholipids compared to control embryos. Phospholipids are important components of the bovine oviductal [33,34,35] and uterine [36,37,38] fluids and may serve as an energy source and as signaling precursors for the gametes and developing embryos. However, there is currently very few information on their roles for the establishment of pregnancy in ruminants. Phospholipids that are regulated in abundance in the oviductal fluid between the periovulatory and luteal stages of cycle are potential candidates for a role in early embryo development. It is noteworthy that molecular species such as PC (34:1), PC (36:4), and PC (36:3) (at m/z 760.59, 782.56, and 784.56, respectively) that were all found more abundant at the periovulatory period than at the luteal stage of the cycle [17] were also identified as overabundant in oEV-exposed blastocysts. Furthermore, several species of PC/SM and PE identified in uterine EVs were reported to be discriminating (by hierarchical clustering) between pregnant and cyclic ewes at day 14, i.e., after blastocyst hatching and elongation [29]. In addition, the PCs detected as increased by pregnancy in the uterine lumen of ewes at day 17, including PC (34:1), PC (34:2), PC (36:1), and PC (36:2) [38], were also found increased in abundance in oEV-exposed blastocysts in the present study.

There is evidence that embryos developed in contact with oviduct epithelial cells, oviduct fluid, and/or oEVs are of higher quality than unexposed controls in terms of morphology, cryotolerance, and gene expression [12,39,40,41]. However, the mechanisms by which oviduct fluid components enhance the quality of developing embryos are poorly known. We previously showed that the steroid hormones progesterone, estradiol, and cortisol added at intraoviductal concentrations to the culture medium altered the phospholipid composition and cryosurvival of bovine embryos [35]. Several phospholipids higher than 700 m/z that were more abundant in embryos exposed to physiological concentrations of steroid hormones—including PC (34:2), PC (36:3), SM (d40:2), PC (36:2), and PC (38:4) at m/z 758.56, 784.56, 785.58, 786.6, and 810.6, respectively—were also found overabundant in blastocysts exposed to oEVs, whereas PC (29:2) or PE (32:2) at m/z 688.43 was found less abundant in both conditions [35]. Two previous studies compared the phospholipid composition of bovine blastocysts conceived in vivo, which have a high likelihood of pregnancy after uterine transfer to a recipient cow; and those produced in vitro, which have lower developmental potential [18,42]. In both studies, the MS data were acquired in the mass range above m/z 700, precluding any comparison below this threshold. However, Sudano et al. [18] evidenced nine phospholipids differentially abundant between in-vivo- and in-vitro-developed bovine blastocysts, among which eight were found more abundant in in-vivo blastocysts. Of note, three PCs (PC (34:2), PC (36:2), and PC (36:1) of m/z 758.56, 786.6, and 788.6, respectively) found here more abundant in oEV-exposed blastocysts were also overabundant among in-vivo-conceived blastocysts [18]. By contrast, in the study from Annes et al. [42], a majority of phospholipids (9/12)—including the molecular species PC (32:1), PC (34:2), PC (38:4)—were absent or less abundant in in-vivo-derived blastocysts than in in-vitro-produced ones, whereas we detected them at higher abundance in oEV-exposed blastocysts or expanded blastocysts than in controls. However, in accordance with Sudano et al. [18], PC (36:2) was more abundant in in-vivo and in-vitro oEV-exposed developed blastocysts [42]. Further studies are now needed to know whether exposure to oEVs in vitro could lead to higher chances of implantation and pregnancy after intrauterine transfer.

In conclusion, this study provides the first analysis of the embryonic lipidome regulated by oEVs during in vitro development. Exposure to oEVs induced significant changes in the phospholipid composition of resulting embryos toward more abundant PC, PE, and SM with long-chain fatty acids. These changes were probably mediated by both the incorporation of oEV phospholipids into embryo membranes and the modulation of embryonic lipid metabolism by other oEV cargos such as mRNAs, miRNAs, and proteins.

## 4. Materials and Methods

### 4.1. In Vitro Embryo Production and oEVs Supplementation

Bovine ovaries were collected at a local slaughterhouse, transported to the laboratory at 32–35 °C, then cumulus oocyte complexes (COCs) were collected by aspirating follicles of 2–8 mm in diameter. The in-vitro maturation (IVM)/in-vitro fertilization (IVF) protocols used in the present study have been described previously [35]. Briefly, groups of 40–50 COCs with more than three compact layers of cumulus cells were matured in 25 mM bicarbonate-buffered TCM 199 supplemented with 10% fetal calf serum (FCS; GIBCO BRL), 10 µg/mL follicle-stimulating hormone (FSH), 10 µg/mL luteinizing hormone (LH; Stimufol; SPRL Reprobiol), 1 µg/mL oestradiol 17β, and 50 µg/mL gentamycin for 22 h at 38.5 °C with 5% CO_2_. Matured oocytes were then fertilized in a modified Tyrode’s bicarbonate buffered solution (fert-TALP; pH 7.6) containing 10 mg/mL heparin sodium salt (H3125; Sigma), 6 mg/mL BSA, 20 mM penicillamine (P4875; Sigma), 10 mM hypotaurine (H1384; Sigma), and 1 mM adrenaline (E4250; Sigma). A single ejaculate from one Normande bull (Evolution cooperative, France) of proven fertility was used in all the IVF experiments. Frozen–thawed Bovipure-Bovidilute (Nidacon)-treated spermatozoa were coincubated with COCs at an insemination dosage of 2 × 10^6^ spermatozoa/mL for 22 h at 38.5 °C in a humidified atmosphere with 5% CO_2_. The day of IVF was defined as Day 0. On Day 1, all presumptive zygotes were denuded and cultured for 8 days in 30 µL of synthetic oviductal fluid (SOF) medium [43] supplemented with 6 g/L BSA under paraffin oil (Origio, CooperSurgical, Trumbull, USA) at 38.5 °C in a humidified atmosphere containing 5% O_2_ and 5% CO_2_.

The oEVs used were obtained from a pool of bovine oviductal fluids collected at a local commercial slaughterhouse and isolated by centrifugation (12,000× *g*, 15 min) and two successive ultracentrifugations (100,000× *g*, 90 min), then characterized by transmission electron microscopy and western blotting as detailed in our previous study [7]. Only oviducts at the postovulatory stage of cycle (recently formed corpus luteum, absence of follicles >10 mm; estimated Days 1–5 postovulation) and ipsilateral to ovulation, i.e., at the expected time and site of embryo development, were used. The same pool of oEVs isolated from 12 postovulatory ipsilateral oviducts was used for Experiments 1–2 and ICM-MS analysis. The oEVs sample was assayed for protein concentration (BCA method), aliquoted in small volumes and stored at −80 °C. In Experiments 1 and 2, oEVs were added to the culture medium at a final concentration of 0.05 mg of proteins/mL, starting on Day 1 with no renewal of the culture medium.

### 4.2. Assessment of Embryo Development and Quality

**Figure 4:**
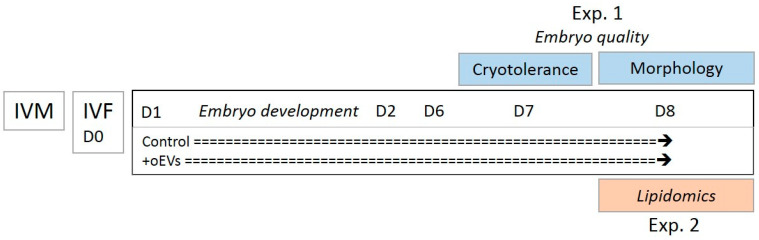
Experimental design.

Two successive experiments were conducted by two teams of operators (including one person in common in the two experiments) in two different IVF laboratories (Experiment 1 in the experimental unit of Allice; Experiment 2 in the PRC unit of INRAE, Nouzilly, France), allowing us to provide enough embryos for the evaluation of developmental rates (Experiments 1 and 2), embryo quality (Experiment 1), and lipidomic profiles (Experiment 2), as summarized in Figure 4. Due to the collection of expanded blastocysts at day 7 in Experiment 1, blastocyst hatching was evaluated only in Experiment 2.

In both Experiments, the number of cleaved embryos was recorded on day 2 and numbers of blastocysts, expanded blastocysts, and hatched blastocysts on days 6, 7, and 8 over 14 replicates in total (7 replicates per Experiment). Expanded blastocysts were distinguished from blastocysts according to the criteria of the International Embryo Transfer Society (IETS; larger blastocoel volume, thinner zona pellucida). In Experiment 1, subgroups of day-7-expanded blastocysts of grade 1 (according to IETS criteria) were submitted to slow freezing and thawing to assess their cryotolerance. Briefly, embryos were washed twice in the commercial Embryo holding medium (EHM, IMV Technologies, L’Aigle, France) then placed by groups of 3–6 in the Embryo freezing medium (EFM, IMV Technologies) containing 1.5 M ethylene glycol and 0.1 M of sucrose for 10 min, then the mixture was placed in a straw. Freezing at 0.3 °C/min was performed up to −32 °C thanks to a Freeze Control cryochamber (Cryologic, Blackburn, Australia), then straws were immersed in liquid nitrogen. For thawing, straws were put in a water bath at 35 °C for 30 s. Embryos were washed for 5 min in EHM then cultured for 2 days in 30 µL of SOF medium supplemented with 1% of postovulatory cow serum and 6 g/L of BSA at 38.5 °C under humidified atmosphere with 5% CO_2_ and 5% O_2_. Numbers of re-expanded and hatched blastocysts were recorded at 24 and 48 h after thawing. Percentages of hatching were calculated from the total numbers of frozen–thawed blastocysts. Four replicates with 3–6 expanded blastocysts per replicate were conducted. In addition, in Experiment 1, blastocysts and expanded blastocysts at day 8 were fixed in phosphate-buffered saline (PBS) containing 4% paraformaldehyde (PBS-PAF 4%, 20 min, 34 °C) for evaluation of total cell numbers under confocal microcopy, as described in detail in our previous study [35]. For statistical analysis, rates of development, blastocyst hatching, and re-expansion/hatching after thawing were compared between groups by Mann–Whitney tests (nonparametric data). Cell numbers per blastocyst were compared by Student t-tests (parametric data).

### 4.3. Lipidomic Profiling of Blastocysts and oEVs by Intact Cell MALDI-TOF Mass Spectrometry

Fresh day-8 blastocysts and expanded blastocysts were washed twice in Tris-sucrose buffer (TSB: 20 mM Tris-HCl, pH 6.8, and 260 mM sucrose) before being individually analyzed for lipidomic profiling by ICM-MS, as described previously [35]. Briefly, using a videomicroscope, individual embryos were immediately spotted onto a plate (Bruker Daltonics, Germany) and the excess of TSB was removed. A total of 0.5 µL of Methanol 100% was deposed on embryo to promote lipid extraction. After evaporation of the methanol, 2 × 0.5 µL of 2,5-Dihydroxybenzoic acid (DHB) matrix at 15 mg/mL dissolved in 90% acetonitrile/9.8% water/0.2% TFA was overlaid on embryos. For oEV analysis, 0.5 µL of oEV sample (at 11.3 mg of proteins per mL) was spotted with 0.5 µL of DHB matrix; a total of six spots were deposited on the MALDI plate. For all samples, the matrix was allowed to evaporate at room temperature for 30 min. Profiles of embryos and oEVs were acquired using a RapifleX Tissuetyper MALDI-TOF instrument (Bruker Daltonics, Bremen, Germany) equipped with a Smartbeam 3D Nd:YAG (355 mm) laser. Each spectrum was obtained in positive and reflector ion modes, at 10-kHz laser repetition rate, with a sampling rate of 1.25 GS s–1, and collected as a sum of 1000 laser shots in 3 shot steps (total of 3000 spectra). Spectra were accumulated in a random walk-on spot within the 350–900 m/z range. A total of 25 blastocysts (16 control, 9 oEV-treated) and 53 expanded blastocysts (28 control, 25 oEV-treated) were analyzed. All embryo and oEVs spots were analyzed in triplicate. External calibration was followed using a mixture of small molecules (1 µL of DHB matrix with 1 µL of calibrant solution containing Caffein, MRFA peptide, LeuEnkephalin, Reserpin, Bradykinin (fragments 2–9), Angiotensin, and Glu-1 Fibrinopeptide B).

### 4.4. Analysis of Lipidomic Data

The analysis of ICM-MS data was performed using the MALDIquant package adapted for mass spectrometry data [44] of the R software (version 3.6.2; free software from the R. foundation), as previously described by our team [17]. To increase mass accuracy (mass error < 0.05%), an internal calibration was subsequently applied to all spectra using flexAnalysis 4.0 software (Bruker, Bremen, Germany) and FlexAnalysis Batch Process (Bruker, Compass 2.0). For all data, a lock mass correction was performed using known PC (34:1) at 760.5851 m/z. The profile spectra were treated for baseline subtraction (SNIP method), smoothing by the Savitzky–Golay algorithm and realignment using prominent peaks and normalization on intensity using the total ion count method. Peaks were detected using a total average spectrum with a signal:background noise > 4. The precision of the ICM-MS acquisitions was determined by calculating the coefficient of variation (CV) from the normalized peak intensity values of the 3–5 technical replicates for each sample. The average CV obtained was 14.6% and 24.8% for analyses on embryos and oEVs, respectively. The MS data did not pass the normality and homogeneity tests. Thus, the nonparametric Kruskal–Wallis test was used for comparison between all groups and the Mann–Whitney test for comparison between treated and control groups at each stage. M/z values were considered differentially abundant between groups with a *p*-value < 0.05. Hierarchical clustering of differential m/z values was performed using Spearman correlations and the gplot package (v 3.0.1.2) of FactoMineR (v 2.1) of the R. software. The differential peaks were annotated based on theoretical masses ± 0.05 m/z using the LIPID MAPS^®^ lipidomics gateway database [45] or using a home databank compiled from the MS identification of lipids in bovine oviductal and follicular fluids [17,46].

## Figures and Tables

**Table 1:** Developmental rates of bovine embryos exposed to oviduct fluid extracellular vesicles (oEVs) during their development in vitro.

No Experiment	Treatment	Number of Cumulus-Oocyte-Complexes (COCs)	Cleaved on Day 2	Blastocyst Yield, N (Mean ± SEM, %)		
			N (Mean ± SEM, %)	Day 6	Day 7	Day 8
Experiment 1	Control	374	318 (85.3 ± 3.2)	10 (3.6 ± 1.1)	67 (21.5 ± 3.8)	105 (33.1 ± 3.8)
	+oEV (treated)	374	316 (85.2 ± 2.4)	18 (4.2 ± 0.6)	85 (27.2 ± 2.8)	115 (40.1 ± 2.6)
Experiment 2	Control	587	468 (80.2 ± 2.0)	21 (4.4 ± 1.4)	77 (15.6 ± 2.8)	85 (20.7 ± 1.8)
	+oEV (treated)	583	484 (83.8 ± 1.7)	20 (4.5 ± 0.9)	67 (15.9 ± 2.4)	65 (17.4 ± 1.9)

## Supplementary Materials

The supplementary materials are available online at https://www.mdpi.com/1422-0067/21/15/5326/s1.

## Abbreviations

COCs	Cumulus-oocyte complexes
ICM-MS	Intact Cell MALDI-TOF—Mass Spectrometry
LPC	Lysophosphatidylcholine
LPE	Lysophosphatidylethanolamine
oEVs	Oviductal extracellular vesicles
PC	Phosphatidylcholine
PE	Phosphatidylethanolamine
SM	Sphingomyelin
