# Supplementary material for: Oviduct Fluid Extracellular Vesicles Change the Phospholipid Composition of Bovine Embryos Developed In Vitro
Source: Int J Mol Sci. 2020 Jul 27;21(15):5326. doi: 10.3390/ijms21155326 (PMC7432015; doi:10.3390/ijms21155326)
Supplement: Supplementary file 1 [file ijms-21-05326-s001.zip › Suppl data B.docx]

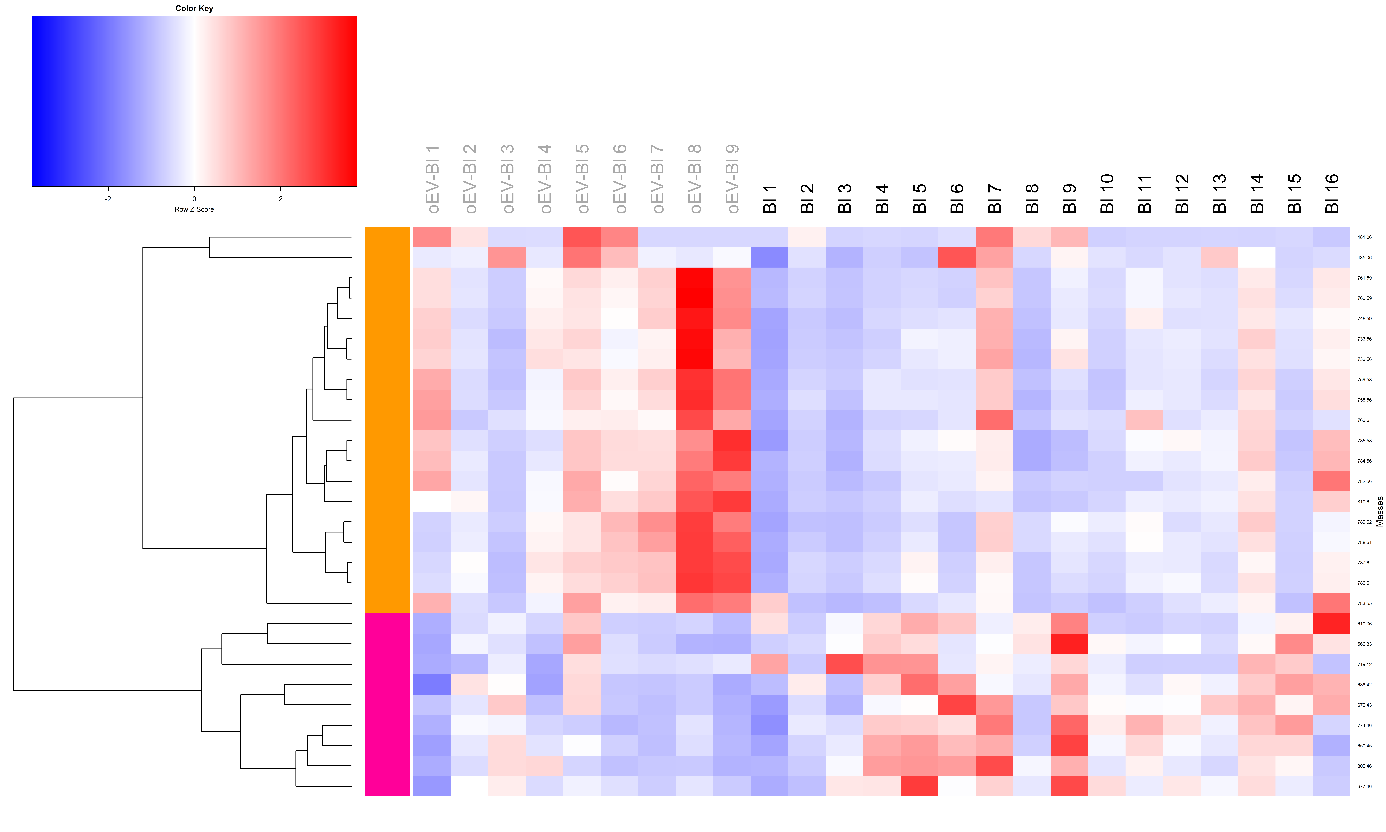


**Figure 1**: Heat map of normalized intensity values obtained by ICM-MS for individual blastocysts.


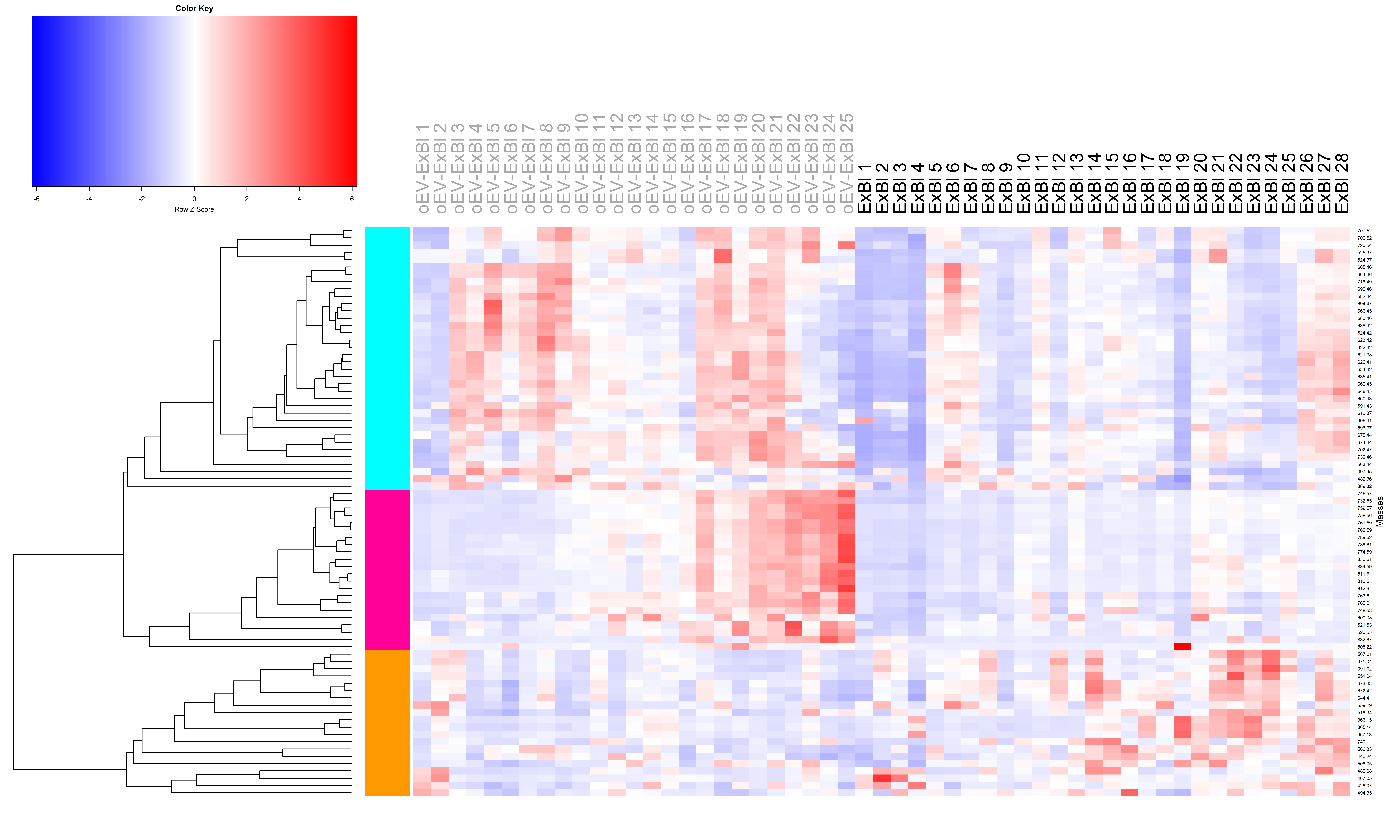


**Figure 2:** Heat map of normalized intensity values obtained by ICM-MS for individual expanded blastocysts.
